# Examining Human-Smartphone Interaction as a Proxy for Circadian Rhythm in Patients With Insomnia: Cross-Sectional Study

**DOI:** 10.2196/48044

**Published:** 2023-12-15

**Authors:** Chen Lin, I-Ming Chen, Hai-Hua Chuang, Zih-Wen Wang, Hsiao-Han Lin, Yu-Hsuan Lin

**Affiliations:** 1 Department of Biomedical Sciences and Engineering, National Central University Taoyuan City Taiwan; 2 Department of Psychiatry, National Taiwan University Hospital Taipei Taiwan; 3 Department of Psychiatry, College of Medicine, National Taiwan University Taipei Taiwan; 4 College of Medicine, Chang Gung University Taoyuan Taiwan; 5 Department of Family Medicine, Chang Gung Memorial Hospital, Taipei Branch and Linkou Main Branch Taoyuan Taiwan; 6 Department of Industrial Engineering and Management, National Taipei University of Technology Taipei Taiwan; 7 Institute of Population Health Sciences, National Health Research Institutes Miaoli County Taiwan

**Keywords:** actigraphy, circadian rhythm, digital biomarkers, human-smartphone interaction, insomnia, intradaily variability, mobile apps

## Abstract

**Background:**

The sleep and circadian rhythm patterns associated with smartphone use, which are influenced by mental activities, might be closely linked to sleep quality and depressive symptoms, similar to the conventional actigraphy-based assessments of physical activity.

**Objective:**

The primary objective of this study was to develop app-defined circadian rhythm and sleep indicators and compare them with actigraphy-derived measures. Additionally, we aimed to explore the clinical correlations of these indicators in individuals with insomnia and healthy controls.

**Methods:**

The mobile app “Rhythm” was developed to record smartphone use time stamps and calculate circadian rhythms in 33 patients with insomnia and 33 age- and gender-matched healthy controls, totaling 2097 person-days. Simultaneously, we used standard actigraphy to quantify participants’ sleep-wake cycles. Sleep indicators included sleep onset, wake time (WT), wake after sleep onset (WASO), and the number of awakenings (NAWK). Circadian rhythm metrics quantified the relative amplitude, interdaily stability, and intradaily variability based on either smartphone use or physical activity data.

**Results:**

Comparisons between app-defined and actigraphy-defined sleep onsets, WTs, total sleep times, and NAWK did not reveal any significant differences (all *P*>.05). Both app-defined and actigraphy-defined sleep indicators successfully captured clinical features of insomnia, indicating prolonged WASO, increased NAWK, and delayed sleep onset and WT in patients with insomnia compared with healthy controls. The Pittsburgh Sleep Quality Index scores were positively correlated with WASO and NAWK, regardless of whether they were measured by the app or actigraphy. Depressive symptom scores were positively correlated with app-defined intradaily variability (β=9.786, SD 3.756; *P*=.01) and negatively correlated with actigraphy-based relative amplitude (β=–21.693, SD 8.214; *P*=.01), indicating disrupted circadian rhythmicity in individuals with depression. However, depressive symptom scores were negatively correlated with actigraphy-based intradaily variability (β=–7.877, SD 3.110; *P*=.01) and not significantly correlated with app-defined relative amplitude (β=–3.859, SD 12.352; *P*=.76).

**Conclusions:**

This study highlights the potential of smartphone-derived sleep and circadian rhythms as digital biomarkers, complementing standard actigraphy indicators. Although significant correlations with clinical manifestations of insomnia were observed, limitations in the evidence and the need for further research on predictive utility should be considered. Nonetheless, smartphone data hold promise for enhancing sleep monitoring and mental health assessments in digital health research.

## Introduction

For decades, actigraphy has served as the standard measurement tool to assess sleep and circadian rhythms. It uses wrist-worn accelerometers to collect data on physical activities, which offer an easy proxy for sleep and circadian rhythm measurements; this includes the time of sleep onset (SO) and wakefulness, as well as circadian rhythm indicators such as the relative amplitude (RA) of the sleep-wake cycle. Although previous systematic reviews and meta-analyses have suggested the central role of physical activity patterns in mood regulation in mood disorders [1-3], the circadian rhythms of mental activities may be more directly related to mood regulation than previously considered. For instance, swiping the smartphone screen can engage mental activities but elicit low-level physical activities below the threshold that actigraphy is able to detect. This condition may be prevalent, especially in patients with insomnia [4]. Such differences between physical and mental activities, as well as the widespread use of smartphones in modern behavior, warrant an updated approach to tracking sleep and circadian rhythms based on mental activity.

Human circadian rhythms can now be observed through digital footprints, which are derived from individuals’ day-to-day interactions with modern technologies, such as smartphones [5]. Several mobile apps were available for automatically measuring sleep using smartphone sensors, including sound, light, movement, screen events, app use, and battery status [6-9]. However, most sleep assessment algorithms demanded high power consumption, and only a few apps used low power through human-smartphone interaction patterns [10-16]. These patterns encompassed 3 types of passive data: screen events [13,15,16], smartphone touchscreen interactions [10,14], and call-detail records [11,12]. Among these, screen events provided comprehensive records of users’ smartphone activities. The time stamps of screen events, which included notifications, screen-on or -off events, and app types, formed time series that delineated human-smartphone interactions and demonstrated good temporal stability [17]. Moreover, these time stamps corresponded to stimuli, individual responses, and the content of stimuli or responses, which are typically measured in a laboratory setting [18]. By leveraging the data recording capabilities of smartphones, these human-smartphone interactions can now be continuously measured in ecologically valid, real-world settings. In our previous research, we used the time stamps of screen events and developed an app-based algorithm, achieving 90.4% accuracy in estimating sleep time [16]. However, it is important to note that our app-based algorithm relied on daily self-reports from a sample of college students and could only identify uninterrupted sleep [16]. Furthermore, none of the previous studies estimating sleep-wake patterns from the time stamps of screen events assessed circadian rhythm indicators such as RA, interdaily stability (IS), and intradaily variability (IV) [10,14]. Additionally, these studies solely focused on healthy participants and lacked validation in clinical settings with individuals experiencing sleep disturbances or disrupted circadian rhythms. As a result, the scarcity of clinical evidence has limited the application of circadian knowledge to diagnosing and treating psychiatric and neurodegenerative disorders. Further research is warranted to validate and refine the use of the time stamps of screen events for assessing circadian rhythms and sleep disturbances, potentially broadening its clinical utility.

In this study, we used standard wrist-worn actigraphy to quantify sleep-wake cycles and, in parallel, recorded the time stamps of human-smartphone interaction patterns. The current version of the “Rhythm” app automatically records smartphone use time stamps and uses an algorithm similar to that of actigraphy to calculate the circadian rhythms of smartphone use. Based on the near-24-hour cycle, we defined app-generated daily sleep indicators and weekly circadian rhythm indicators. We hypothesized that the indicators derived from human-smartphone interaction patterns and those derived from actigraphy recordings represented the circadian rhythms of mental activities and physical activities, respectively. Furthermore, it was hypothesized that clinical outcomes would be more strongly correlated with app-defined indicators than with actigraphy indicators. This study aimed to (1) develop app-defined circadian rhythm and sleep indicators, (2) compare these indicators with actigraphy-derived measures, and (3) investigate the clinical correlation between these circadian rhythm and sleep indicators and self-reported depressive symptoms and sleep quality in individuals with insomnia and healthy controls.

## Methods

### Study Design

This cross-sectional study was designed with 2 primary objectives: first, the development of app-defined circadian rhythm and sleep indicators, and second, a comprehensive comparison and contrast of these novel indicators with their counterparts derived from standard actigraphy measures. To fulfill these aims, we created the “Rhythm” mobile app to capture smartphone use time stamps and transform them into sleep and circadian rhythm indicators. The research involved two distinct participant groups: (1) a total of 33 individuals diagnosed with insomnia and (2) an age- and gender-matched control group of 33 healthy participants. Over a minimum span of 4 weeks, we used standard wrist actigraphy to measure participants’ sleep-wake patterns.

The study protocol consisted of several analytical components. We initially conducted an examination to uncover any discrepancies between the app-defined circadian rhythm and sleep indicators and their corresponding actigraphy-derived counterparts. Subsequently, we carried out analyses to compare these indicators between the patients with insomnia group and the healthy control group. This study delved into clinical associations by investigating correlations between sleep indicators, measured by the app or actigraphy, and self-reported sleep quality. Additionally, we explored clinical associations by examining correlations between circadian rhythm indicators, measured by the app or actigraphy, and self-reported depressive symptom scores.

In determining the optimal sample size for this study, we considered 2 fundamental objectives. The first was to compare app-defined circadian rhythm and sleep indicators with measures derived from actigraphy, requiring an analysis at the person-days level. The second objective aimed to investigate correlations between specific indicators and self-reported depressive symptoms, as well as sleep quality, among individuals with insomnia and healthy controls.

To guide our determination of sample size, we drew insights from pertinent studies in related areas [19-22]. These studies explored the daytime sleep-tracking performance of commercial wearable devices at home [19], the reproducibility of a standardized actigraphy scoring algorithm for sleep in a US Hispanic or Latino population [22], the validation of consumer sleep wearable devices with actigraphy and polysomnography in adolescents [20], and the comparison of consumer sleep-tracking devices with polysomnography [21]. The participant counts in these studies ranged from 16 to 58, and the data collection spanned from 112 to 350 person-days [19-22].

Our selected sample size of 66 individuals effectively addresses both our comparison and correlation objectives. This size aligns with, or even exceeds, those found in the previously mentioned studies. Importantly, this sample size equips us with ample statistical power to explore complex relationships among multiple variables within our analytical framework. The inclusion of diverse studies bolsters the strength of our findings.

Regarding the investigation of clinical correlations, the dependent variables encompass self-reported depressive symptoms or self-reported sleep quality. The most critical independent variable involves circadian rhythm or sleep indicators, followed by age, gender, and an additional variable representing physical activity level or smartphone use. Abiding by the “one in ten rule,” which offers a guideline for predictor parameters in regression analysis [23,24], our sample size of 66 individuals is adequate to accommodate up to 4 independent variables, as necessitated by our analytical framework. This estimation is supported by the fact that a sample size of over 40 is required based on the above considerations.

### Participants

Between October 2019 and September 2022, a total of 33 individuals diagnosed with insomnia (24 female individuals; mean age 45.3, SD 14.1 y) and an age- and gender-matched control group of 33 healthy participants (24 female individuals; mean age 42.5, SD 9.1 y) were recruited for this study. The age range for participants was confined to individuals aged between 20 and 65 years.

For the patients with insomnia group, participants were required to express willingness to engage in the study and align with specified research prerequisites, including possessing access to the requisite smartphone app. The diagnosis of insomnia adhered to specific criteria outlined in the *Diagnostic and Statistical Manual of Mental Disorders, Fifth Edition* (DSM-5) and was conducted through in-depth interviews by experienced psychiatrists. Notably, all of the patients with insomnia exhibited comorbid anxiety or mood disorders and were under treatment with sedatives or hypnotics, receiving care from psychiatrists YHL and IMC at 2 outpatient clinics.

The healthy control group was recruited by selecting 33 participants from a cohort of medical staff actively engaged in a workplace health promotion initiative across 7 hospitals in northern Taiwan. Recruitment was carried out through various channels, including email, intranet announcements, and posters within the hospital premises. This approach ensured a one-to-one match with the recruited patients with insomnia. The selection of healthy controls was guided by age and gender considerations to ensure alignment with the patients with insomnia group. The healthy controls did not meet the criteria for insomnia according to the DSM-5. Notably, none of the medical staff members were excluded from night shifts during the study duration, promoting a more comprehensive assessment and minimizing potential biases concerning sleep patterns. Recruitment procedures were uniformly executed for both the patients with insomnia group and the healthy controls, with physicians approaching potential participants meeting the study’s inclusion criteria. In parallel, research assistants engaged potential healthy controls, ensuring consistency across recruitment strategies.

All eligible participants expressing interest underwent a comprehensive informed consent process before inclusion. Following informed consent, participants were requested to install the app and wear a wrist-mounted actigraphy device for a minimum of 4 weeks. This combined effort contributed to a data set comprising 2097 person-days of data collection. Participation in the study was restricted to individuals possessing Android smartphones due to technical constraints associated with the research tools. To ensure data integrity, participants were explicitly instructed not to share their smartphones with others during the study duration. Although participants were unaware of the specific analysis linking actigraphy with the app until the study’s conclusion, they wore the wrist actigraphy devices throughout the entire observation period. Worth noting is that while participants were instructed to wear their wrist actigraphy devices consistently, specific guidance concerning smartphone placement was not provided.

### Mobile App “Rhythm” and Actigraphy

#### Actigraphy Measurement

Participants were instructed to wear the research-grade wrist actigraphy device on their nondominant wrist for a minimum of 4 weeks. Accelerations along 3 axes were gathered by the actigraphy watches, combined using the Euclidean distance of the accelerations’ deviation from zero (Z) and a bandpass filtered from 0.5 to 3 Hz. The Z values over a predefined threshold are integrated within 2 seconds, and the activities of 1-second epochs (acti-counts) are derived from averaging the integrated segments within 1 minute [25,26].

We used the standard Cole-Kripke algorithm with minor adaptations to estimate putative sleep and wake times (WTs) from the acti-count data. This algorithm categorizes data into rest-active states on a minute-by-minute basis, using a weighted sum of the current minute with contiguous minutes to mitigate the influence of sudden fluctuations in activity that could potentially affect categorization accuracy. The implementation of the algorithm was conducted using MATLAB (MathWorks), using preexisting codes. By integrating both objective actigraphy data and subjective participant input, our intention was to enhance the reliability and accuracy of actigraphy as a sleep measurement tool.

Acknowledging the significance of actigraphy validation studies against established polysomnography, which includes the use of actigraphy alongside a sleep diary and a standardized scoring protocol [22], we adapted a comparable approach to validate our actigraphy-derived sleep times. Given that all participants were required to install the mobile app “Rhythm” and wear a wrist actigraphy device for at least 4 weeks, a significantly longer period than the study using a standardized scoring protocol where participants underwent 7 days of continuous wrist actigraphy and completed daily sleep diaries, we introduced an additional step involving weekly phone interviews with participants, similar to a sleep diary approach, to cross-reference and corroborate the actigraphy data.

#### Smartphone Use Measurement

**Figure 1:**
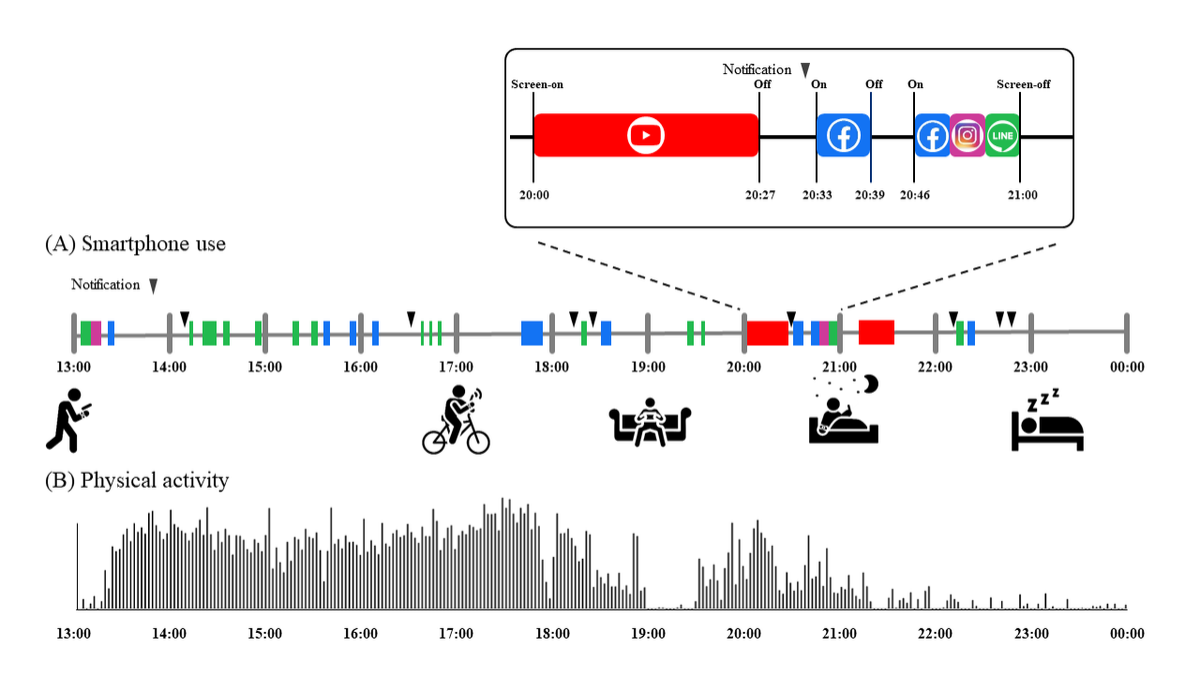
The study design. (A) The app, Rhythm, automatically recorded smartphone events as 3 key variables: time stamp of the screen-on or -off events, notification (inverted triangle), and label of the app in use. The box at the top right corner shows the details of the smartphone events. (B) We used standard wrist-worn actigraphy to quantify sleep-wake cycles and, in parallel, recorded the timestamps of human-smartphone interaction patterns.

The app automatically recorded smartphone events as 3 key variables: time stamp of screen-on or -off events, time stamp of notifications, and label for the app in use [13,16]. To accurately capture app use behaviors in real-life scenarios, we considered the possibility of users temporarily leaving their smartphones, such as during work, charging, or other activities, while they remain active. This intermittent disengagement from the smartphone poses a challenge in accurately representing app use patterns. To address this issue, we introduced the “app-count” method, which involves using longer durations to represent app use behaviors (Figure 1). Specifically, the “app-count” was defined as the sum of minute-by-minute use counts in nonoverlapping 5-minute epochs (288 epochs/d). By using longer durations, we aimed to encompass the overall engagement with the smartphone during both active use and periods of temporary disengagement.

**Figure 2:**
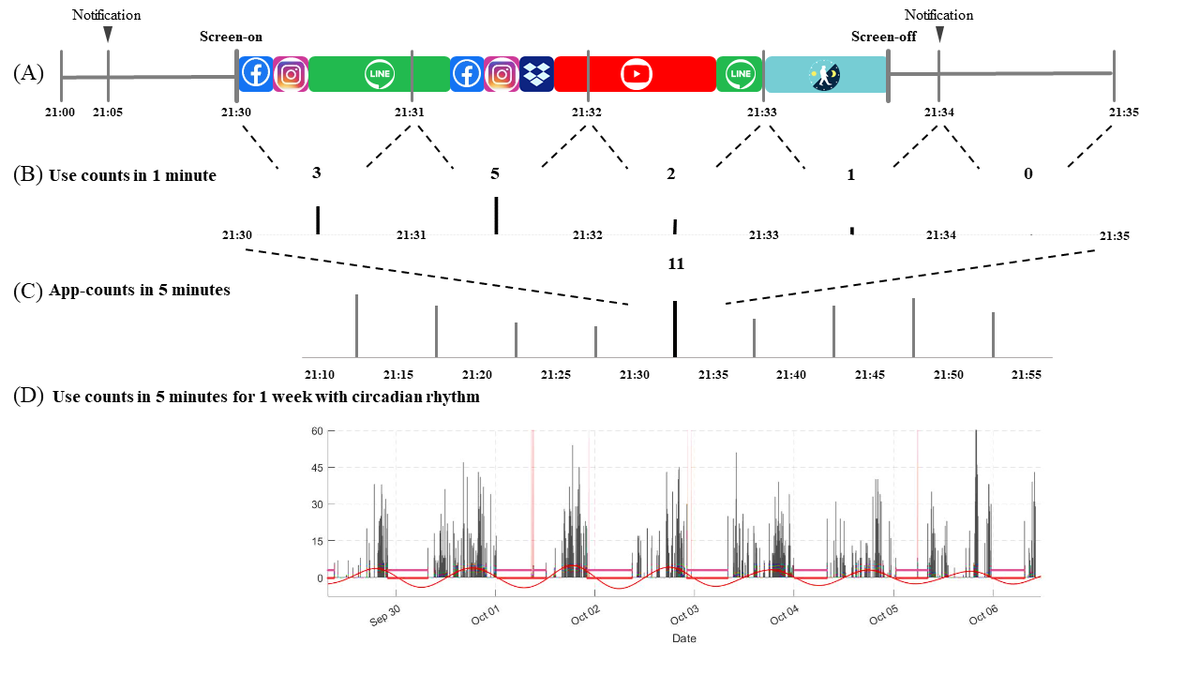
The algorithm of the smartphone use count. (A) Smartphone use from screen-on to the successive screen-off was defined as 1 episode. (B) The use counts were defined as the number of app uses per minute. (C) The app-count was defined as the minute-by-minute use counts summed up into a 5-minute epoch to avoid excessive zero-count segments due to the nature of the use data. (D) We used the single-component single cosinor model to fit the time series of app-counts. In this study of circadian rhythm, it is reasonable to assume that the period is known, being synchronized to the 24-hour cycle. The 24-hour cycle comprises an active phase, including the acrophase, and an inactive phase, including the nadir. Circadian rhythm indicators quantify the regularity, shape, and timing of these app-counts. We further identified sleep time during the inactive phase by the threshold of app-use counts.

Figure 2 illustrates the following steps: (1) to quantify the “app-count” of smartphone use in a 5-minute time window by considering app-count as the number of switches from one app to another per minute, noting that herein app-count was used to mimic acti-count in actigraphy; (2) to determine the near-24-hour cycle (ie, the circadian rhythm), which comprises an active phase, including the acrophase, and an inactive phase, including the nadir; and (3) to identify sleep time during the inactive phase by the threshold of app-counts.

#### Circadian Rhythm and Sleep Indicators

The acti-counts and app-counts generated from smartphone use data were processed into 6 sleep indicators and 3 circadian rhythm indicators.

**Figure 3:**
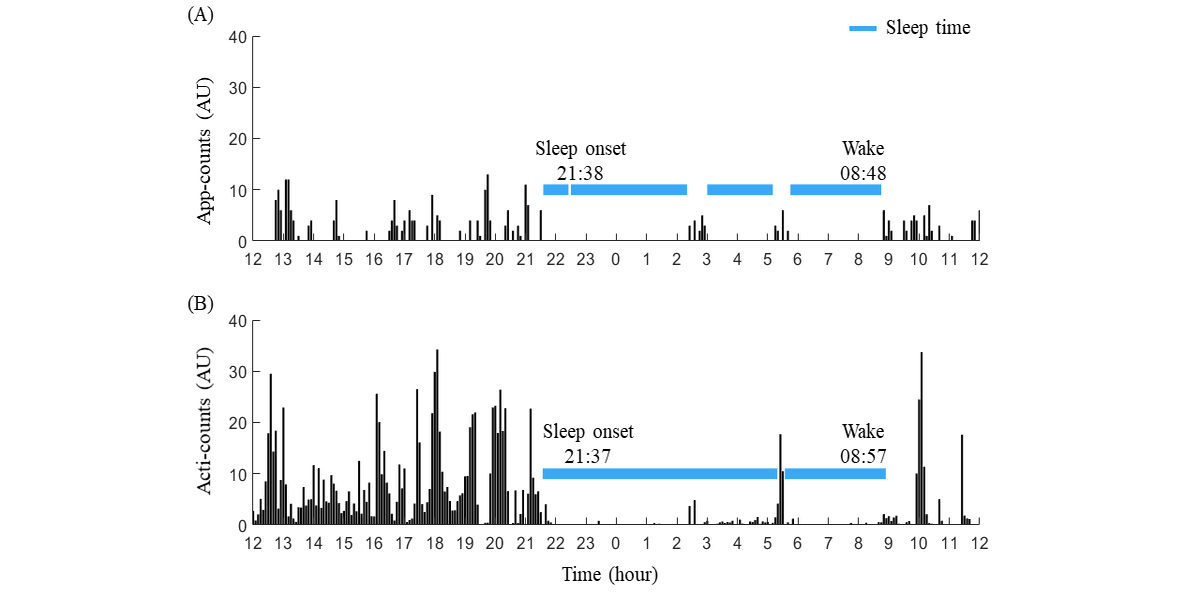
The sleep indicators derived from smartphone use and physical activity. (A) The sleep indicators were computed based on the 5-minute epoch of the app-count. The approximately 16- to 24-hour-long cycles of app-count were extracted by a band-pass filter, and putative sleep was assumed to occur in the inactive phase with nadir. The time with 8 consecutive epochs (40 minutes) with 0 app-count was considered sleep onset (SO), and the time with 6 consecutive epochs (30 minutes) with nonzero app-count was considered wake time. The wake after sleep onset (WASO) was calculated by the number of epochs with a nonzero app-count after SO. There were 3 WASOs (ie, the number of awakenings [NAWK] was 3) and the duration of the WASO was 95 minutes. We also measured the SO (3:13) and the wake time (8:48). The total sleep time (TST) was 575 minutes. (B) The actigraphy-defined sleep indicators showed a similar actigraphy-based sleep onset (SOact) of 21:37, actigraphy-based wake time of 8:57, a shorter app-defined wake after sleep onset (WASOapp) of 10 minutes, and a longer app-defined total sleep time of 670 minutes than their app-defined counterparts. AU: arbitrary unit.

The daily sleep indicators included SO, WT, the midpoint of sleep, wake after sleep onset (WASO), total sleep time (TST), and the number of awakenings (NAWK). To better estimate the daily sleep indicators from the app-count, we extracted the approximately 16- to 24-hour-long cycles by band-pass filter, and putative sleep was assumed to occur at the half-cycle with nadir. The time with 8 consecutive epochs with zero app-count was considered SO, and the time with 6 consecutive epochs with nonzero app-count was considered WT. The WASO was calculated by the number of epochs with a nonzero app-count after SO (Figure 3).

**Figure 4:**
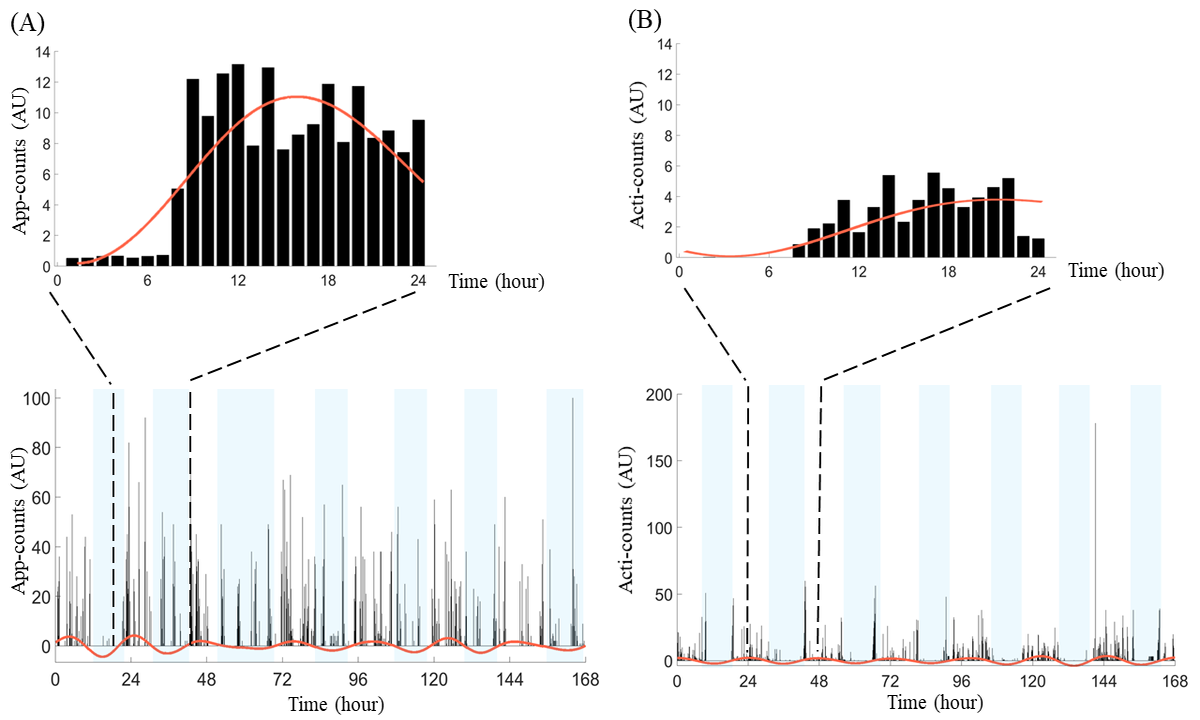
The circadian rhythm indicators were calculated from at least 1-week-long data sets of (A) app-counts or (B) acti-counts. The nonparametric method was used to calculate 3 circadian rhythm indicators: relative amplitude (RA), interdaily stability (IS), and intradaily variability (IV). RA reflected the difference in activity levels between the most and least active periods of the day. IS represents the degree of consistency of activity patterns from one 24-hour period to the next. IV quantified the fragmentation between periods of activity and periods of rest within a 24-hour period. The app-defined circadian rhythm indicators showed significantly higher RA, lower IS, and higher IV than their actigraphy-defined counterparts. AU: arbitrary unit.

The derived parameters were applied to the actigraphy in many different patient groups; we also adopted them for our app-count data to quantify human-smartphone interaction patterns. We used a nonparametric method to calculate circadian rhythm indicators based on the 4-week data set of app-counts or acti-counts (Figure 4). The nonparametric method was used to calculate 3 of the circadian rhythm indicators: RA, IS, and IV.

RA, the ratio of the differences between the most active 10 continuous hours (M10) and the least active 5 continuous hours (L5) over the summation of M10 and L5, was calculated to measure the amplitude of rest-activity rhythms while considering the daily variations and unbalanced amplitudes of peak and trough in daily activity or app-use rhythms.

IS quantified the stability of the rhythms between days, that is, the coupling strength of the rhythms to the supposedly stable environmental factors. It could vary between 0 and 1, with higher values indicating more stable daily rhythms.

IV indicated the fragmentation of the rhythms, that is, the frequency and extent of transitions between rest and activity. It could vary roughly between 0 and 2, with higher values indicating higher fragmentations.

RA, IS, and IV were calculated for a minimum period of 1 week [27].

This study used all 3 nonparametric indicators rather than the indicators through cosinor (parametric) analysis. The units of most indicators of cosinor analysis are app-counts or acti-counts, so those indicators are not comparable.

### Self-Reported Questionnaires on Sleep Quality and Depressive Symptoms

Participants’ sleep quality and depressive symptoms were evaluated at the end of the study using self-administered questionnaires, namely the Pittsburgh Sleep Quality Index (PSQI) and the Patient Health Questionnaire (PHQ-9).

#### PSQI Measurement

The PSQI was used to measure the overall sleep quality of individuals in 1 month. This index comprises 19 items that evaluate 7 components of sleep quality, and the sum of the 7 component scores yields 1 total score of subjective sleep quality (range 0-21); higher scores represent poorer subjective sleep quality. The cutoff score for PSQI-defined cases of poor sleep quality is a 6 or greater [28]. A Taiwanese version of the PSQI had been validated with adequate reliability [29], and the Cronbach α in this study was .802.

#### PHQ-9 Measurement

The PHQ-9 is a self-administered questionnaire for depressive symptoms. Each of the 9 depressive symptom criteria yields a score between 0 and 3, such that the PHQ-9’s total score ranges from 0 to 27. A PHQ-9 score of 10 or greater has a sensitivity of 93% and a specificity of 88% for the diagnosis of a major depressive episode [30]. The diagnostic validity of the Chinese version of the PHQ-9 is comparable with clinician-administered assessments [31], and the Cronbach α in this study was .924.

### Statistical Analysis

In this study, we used a 2-way ANOVA to compare the app-defined circadian rhythm and sleep indicators with their actigraphy counterparts. Additionally, we investigated the differences in these indicators between patients with insomnia and healthy controls using the same statistical approach.

To assess the clinical relevance of the sleep indicators measured by the “Rhythm” app, we conducted multivariate regression models to explore their association with subjective sleep quality, represented by the total PSQI scores. Specifically, we focused on understanding how 2 sleep indicators, namely WASO and NAWK, obtained from both the app and actigraphy data, related to participants’ PSQI scores. In these analyses, we controlled for potential confounding factors such as age and gender.

To examine the relationship between circadian rhythm indicators (IS, IV, and RA) and PHQ-9 scores, a measure of depressive symptoms, we used multivariate regression models. Within these models, we designated PHQ-9 scores as the dependent variable, while the circadian rhythm indicators (IS, IV, and RA) were introduced as independent variables. These analyses also encompassed the control of potential confounding factors, including age and gender. It is noteworthy that previous research [32] has highlighted significant variations in rest-activity rhythm patterns based on sex and age. Furthermore, we used an additional model to account for potential confounding effects. In this model, we incorporated age, gender, and 1 of 3 supplementary independent variables: overall activity level, overall smartphone use, or nighttime phone use. This comprehensive approach ensured that we addressed and mitigated potential confounding factors while investigating the relationship between circadian rhythms and depressive symptoms.

A value of *P*<.05 was considered to be statistically significant. Data arrangement and statistical analysis were performed using SPSS Statistics (version 25; IBM Corp).

### Ethical Considerations

The study protocol received approval from the institutional review boards of the National Taiwan University Hospital (202004005RIND) and the Chang-Gung Memorial Hospital (202002452A3 and 202100434B0A3). The study was conducted in accordance with the principles outlined in the Declaration of Helsinki. Prior to their inclusion in the study, participants provided informed consent. To safeguard the privacy and confidentiality of participants, all collected data underwent rigorous de-identification processes before analysis. It is important to note that participants were not subjected to any compensation obligations related to their participation in the study.

## Results

### Participant Characteristics

A total of 33 patients with insomnia had higher PSQI scores (mean 10.2, SD 4.6 vs mean 4.4, SD 2.1; *P*<.001) and depressive symptom scores (mean 10.6, SD 6.6 vs mean 1.8, SD 2.8; *P*<.001) than the 33 healthy controls. There were no significant differences in age (*P*=.17) and gender proportion between the 2 groups (*P*>.99).

### Comparison of Circadian Rhythm and Sleep Indicators

**Figure 5:**
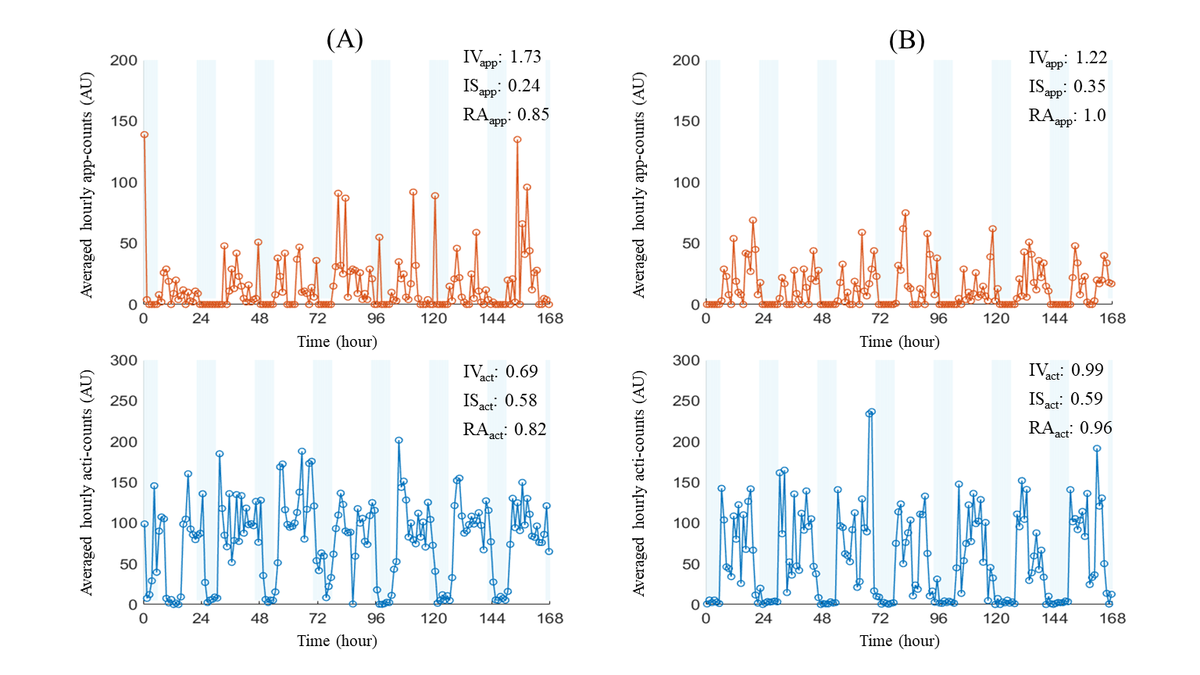
Circadian rhythms of (A) patients with insomnia and (B) healthy controls were measured using both actigraphy and the Rhythm app. The left panel depicts a representative patient with insomnia (Pittsburgh Sleep Quality Index: 10 and Patient Health Questionnaire: 21), while the right panel portrays a healthy control participant (Pittsburgh Sleep Quality Index: 1 and Patient Health Questionnaire: 0). The upper graph illustrates the smartphone use data measured over 7 days using the app, indicated in orange, while the lower graph shows the activity data measured by actigraphy during the same time frame, shown in blue. Although visual inspection of the graphical representation suggests that circadian rhythms of patients with insomnia, whether measured by actigraphy or the app, appear more irregular than those of healthy controls, quantified circadian rhythm indicators reveal consistency with our research findings: patients with insomnia exhibit higher app-defined intradaily variability (IVapp) and lower actigraphy-based intradaily variability (IVact), alongside lower actigraphy-based relative amplitude (RAact). AU: arbitrary unit; ISact: actigraphy-based interdaily stability; ISapp: app-defined interdaily stability; RAapp: app-defined relative amplitude.

Table 1 and Figure 5 show the comparison of app-defined and actigraphy-defined circadian rhythm and sleep indicators. Patients with insomnia and healthy controls both presented (1) no significant differences between app-defined SO (SO_app_) and actigraphy-based SO (SO_act_; *P*=.92), (2) no significant differences between app-defined WT (WT_app_) and actigraphy-based WT (WT_act_; *P*=.34), and (3) an 8.7 (SD 41.4)-minute longer app-defined WASO (WASO_app_) than actigraphy-based WASO (WASO_act_). Finally, there was no significant difference between app-defined TST (TST_app_) and actigraphy-based TST (TST_act_; *P*=.74).

**Table 1:** The comparison of app-defined and actigraphy-based circadian rhythm and sleep indicators in patients with insomnia and healthy controls.

Variable		Patients with insomnia, mean (SD)		Healthy controls, mean (SD)			*P* value differences		
		App	Actigraphy	App	Actigraphy	Group^a^		Measurement^b^	Interaction^c^
**Sleep indicators**									
	Sleep onset^d^	23.80 (1.39)	23.98 (1.37)	23.56 (0.91)	23.34 (0.80)	.03		.92	.34
	Wake time^d^	7.91 (1.41)	7.73 (2.02)	7.12 (0.85)	6.85 (0.79)	.001		.34	.86
	Midpoint of sleep^d^	3.86 (1.30)	3.85 (1.53)	3.34 (0.71)	3.10 (0.69)	.001		.53	.55
	Number of awakenings	0.55 (0.61)	0.66 (0.53)	0.37 (0.37)	0.26 (0.26)	<.001		.99	.17
	Wake after sleep onset (minutes)	18.44 (19.39)	12.17 (17.56)	12.95 (15.09)	2.74 (2.61)	.005		.002	.46
	Total sleep time (minutes)	468.09 (60.99)	452.95 (103.36)	440.96 (57.21)	447.85 (46.53)	.19		.74	.37
**Circadian rhythm indicators**									
	Relative amplitude	0.93 (0.08)	0.81 (0.09)	0.95 (0.06)	0.90 (0.08)	<.001		<.001	.005
	Interdaily stability	0.24 (0.11)	0.40 (0.16)	0.21 (0.09)	0.45 (0.07)	.70		<.001	.03
	Intradaily variability	1.23 (0.23)	0.91 (0.24)	1.13 (0.20)	1.04 (0.26)	.66		<.001	.005

^a^The effect of patients with insomnia versus health controls.

^b^The effect of app versus actigraphy.

^c^The interaction effect of group and measurement.

^d^Time are in day decimal time, for example, 23.50=23:30 PM.

### Associations Between Sleep Indicators and Subjective Sleep Quality

Both app-defined and actigraphy-defined sleep indicators showed later SO (*P*=.03) and WT (*P*=.001) in patients with insomnia than in healthy controls, with statistical significance. Patients with insomnia showed a greater NAWK and a longer WASO than healthy controls (Table 1). Table 2 shows that the PSQI scores were positively associated with WASO_app_, WASO_act_, app-defined NAWK (NAWK_app_), and actigraphy-based NAWK (NAWK_act_) after controlling for participants’ age and gender.

**Table 2:** Comparisons of associations between subjective sleep quality, depressive symptoms, and objective circadian rhythms involving sleep indicators defined by app and actigraphy.

Variable			App								Actigraphy							
			Coefficients (SE)		2-tailed *t* (*df*=62)		*P* value			Coefficients (SE)				2-tailed *t* (*df*=62)			*P* value	
**Sleep indicators versus sleep quality**								.002										<.001
	Number of awakenings	3.327 (1.015)		3.278					4.211 (1.142)						3.688			
	Wake after sleep onset (minutes)	0.096 (0.029)		3.281					0.153 (0.040)						3.843			
**Circadian rhythm versus depressive symptoms**																		
	Relative amplitude	–3.859 (12.352)		–0.312		.76			–21.693 (8.214)				–2.641			.01		
	Interdaily stability	1.578 (8.124)		0.194		.85			–12.003 (6.320)				–1.899			.06		
	Intradaily variability	9.786 (3.756)		2.605		.01			–7.877 (3.110)				–2.533			.01		

Sleep quality was measured by the PSQI, and depressive symptoms were measured by the PHQ-9. Coefficients (β) represent estimates of PSQI or PHQ-9 scores in multivariate regression models. PSQI scores were examined in relation to sleep indicators (WASO and NAWK), while PHQ-9 scores were studied concerning circadian rhythm indicators (IS, IV, and RA). Both analyses controlled for age and gender. The *P* value is used in the comparison of coefficients involving the app and actigraphy.

### Associations Between Circadian Rhythm Indicators and Depressive Symptoms

**Figure 6:**
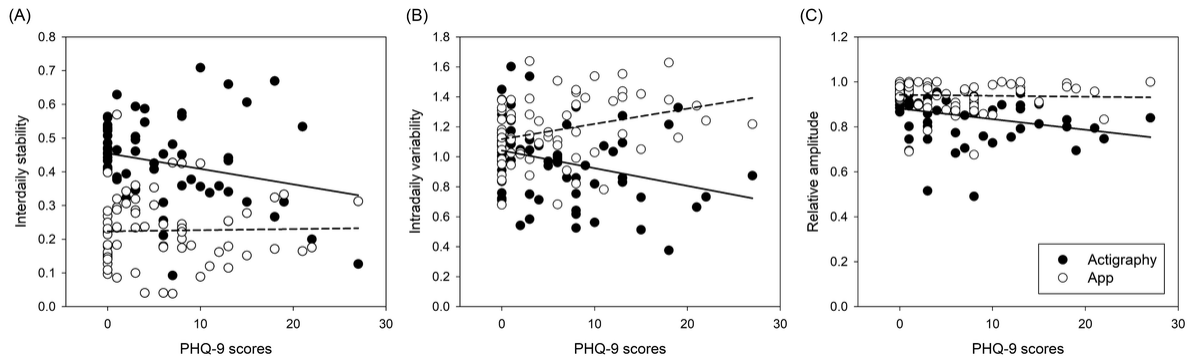
The correlations between depressive symptom scores and circadian rhythm indicators: (A) interdaily stability, (B) intradaily variability, and (C) relative amplitude. The depressive symptoms were measured by the total scores of the Patient Health Questionnaire-9 (PHQ-9). The hollow circles indicate an individual’s depressive symptoms and the value of circadian rhythm indicators measured by the app, and the dashed line indicates the regression line. Meanwhile, the solid circles indicate the actigraphy counterparts, and the black solid line indicates the regression line.

Table 2 and Figure 6 present the results of our analyses, which aimed to explore the associations between depressive symptom scores and various circadian rhythm indicators while controlling for age and gender. The findings revealed significant correlations between depressive symptom scores and certain indicators. Specifically, app-defined IV (IV_app_) showed a positive association with depressive symptoms (β=9.786, SD 3.756; *P*=.01), while actigraphy-based IV (IV_act_; β=–7.877, SD 3.110; *P*=.01) and actigraphy-based RA (RA_act_; β=–21.693, SD 8.214; *P*=.01) exhibited negative associations.

Upon recognizing the potential influence of confounding factors on the relationship between lower IV_act_ and higher depression symptoms, we conducted additional analyses. To account for participants’ overall activity level, represented by the M10 value derived from actigraphy data, we included it as an independent variable in addition to age and gender. The M10 value is a widely accepted indicator of a person’s overall physical activity level [33,34]. Our examination revealed a negative correlation between IV_act_ and the M10 value (*r*=–0.621; *P*<.001) measured by actigraphy. Even after adjusting for overall activity level (M10 value), the correlation of lower IV_act_ with higher depression symptoms remained significant (β=–13.061, SD 3.857; *P*=.001).

Similarly, we further investigated the potential relationship between phone use and mood based on intriguing findings from our sample. To explore this association, we assessed the correlations between IV_app_ and indicators of overall phone use or nighttime phone use. These indicators included overall screen-on time and app-switch frequency, representing phone use. Nighttime phone use was defined as the period between 22:00 and the next 6:00. Our analysis revealed a negative correlation between IV_app_ and overall smartphone use time (*r*=–0.416; *P*<.001) and overall app-switch frequency (*r*=–0.396; *P*<.001). However, there were no significant correlations between IV_app_ and nighttime smartphone use time (r=–0.220; *P*=.08) or nighttime app-switch frequency (*r*=–0.237; *P*=.06).

To further elucidate the relationship, we included these indicators of overall phone use or nighttime phone use, in addition to age and gender, as independent variables in our analysis. The results indicated that the correlation of higher IV_app_ with higher depression symptoms remained significant even after controlling for overall smartphone use time (β=9.102, SD 4.066; *P*=.03), overall app-switch frequency (β=9.366, SD 4.021; *P*=.02), nighttime smartphone use time (β=9.637, SD 3.841; *P*=.02), or nighttime app-switch frequency (β=9.694, SD 3.848; *P*=.01).

## Discussion

### Principal Findings

In this study, we investigated circadian rhythm and sleep indicators in individuals with insomnia, a population that had received limited attention in previous smartphone-based sleep-wake cycle estimations. To achieve this objective, we adopted a novel approach, capturing circadian rhythms derived from human-smartphone interactions, which complemented the physical activity-based circadian rhythms obtained from actigraphy. Our findings revealed significant correlations between the IV_app_ and depressive symptom scores, as well as between the RA_act_ and depressive symptom scores. These correlations aligned with existing patterns observed in individuals with depression [35-38], suggesting disrupted circadian rhythmicity in this population. On the other hand, we observed negative correlations between the IV_act_ and depressive symptom scores, while no significant correlation was found between the app-defined RA (RA_app_) and depressive symptoms. It is important to emphasize that IV was not a direct measure of circadian rhythms per se; rather, it represented the fragmentation in activity profiles between daily rest and activity periods [39]. The different correlations between depressive symptoms and circadian rhythm indicators, as measured by the app and actigraphy, may have reflected varying clinical implications and applications of circadian rhythms based on different types of activities. For instance, actigraphy primarily captured physical activity, while the app-derived indicators might have been associated with activities beyond the physical aspects, which could be interpreted as potential mental activity.

### Comparison to Previous Work

Previous studies on smartphone-based sleep-wake cycle estimations primarily focused on healthy participants and used experimental sleep disruption protocols to evaluate device algorithm performance [10-16,21]. In contrast, this study took a unique approach by specifically examining individuals with insomnia, contributing to the understanding of sleep interruptions, which manifest as WASO, TST, and the NAWK in clinical settings. Moreover, our algorithm was designed to theoretically correspond more closely to mental activities than the tappigraphy algorithm [10], as certain smartphone touchscreen interactions might be less consciously engaged [40]. This study revealed that WASO_app_ was longer than WASO_act_, indicating that human-smartphone interactions while users were still in bed could be detected as periods of wakefulness, even when physical activity fell below the accelerometer’s threshold for identifying wakefulness. This discrepancy aligned with the common underestimation of WASO by actigraphy and other consumer devices in previous studies [21]. Therefore, our findings suggest that WASO_app_ may serve as a potential digital biomarker to capture disturbed sleep, complementing the clinical implications of WASO_act_. These observations are consistent with the findings of tappigraphy, which demonstrated that the probability of touches on smartphones remained greater than 0 for approximately 2 hours after SO_act_ [10].

Furthermore, our findings were consistent with the existing literature, showing an association between RA_act_ and depressive symptoms [41]. However, we did not observe a significant association between RA_app_ and depressive symptom scores, partly due to the invariant nature of RA_app_ resulting from specific app-defined least active period (L5_app_). This was reflected in the higher and invariant nature of RA_app_, compared to RA_act_, with the L5_app_ approaching 0. This implies that there was minimal smartphone use during the L5_app_, while detectable physical activity was still recorded during the actigraphy-defined least active period. Overall, this study builds upon previous research and provides important insights into the potential applications and limitations of smartphone-based assessments for individuals with insomnia.

### Strengths

This study offered several strengths that contributed to the understanding of smartphone-based circadian rhythm and sleep indicators, particularly in individuals with insomnia. One of the notable strengths was the unique focus on patients with insomnia, a population that had received limited attention in previous smartphone-based sleep-wake cycle estimations. By examining sleep disruptions and circadian rhythm indicators in this specific group, our findings provided valuable insights into the clinical relevance of these indicators in real-world settings. Another strength lay in our novel approach of capturing circadian rhythms derived from human-smartphone interactions, which complemented the physical activity–based circadian rhythms obtained from actigraphy. This methodological innovation opened up new avenues for exploring mental activity-based circadian rhythms, allowing us to gain a deeper understanding of potential associations between smartphone interactions, circadian rhythms, and mental health indicators. The observed correlations between IV_app_ and depressive symptom scores, as well as RA_act_ and depressive symptom scores, offered preliminary evidence of the relevance of smartphone-derived circadian rhythm indicators in mental health research. Furthermore, this study highlighted the potential of smartphone apps in digital health research and applications. With the increasing ubiquity of smartphones and the continuous development of health-related apps, our research demonstrated the feasibility of using smartphones as a convenient tool to gather data on circadian rhythms and sleep patterns. The use of smartphone apps for digital phenotyping and health monitoring held promise for future medical research, particularly in understanding the impact of sleep disruptions and circadian rhythm disturbances on mental health outcomes. In addition to its clinical applications, the smartphone app developed in this study could serve as a valuable tool for internet medical research. As data from smartphone interactions and app use could be easily collected remotely and in real time, researchers could harness this information to conduct large-scale epidemiological studies or gather longitudinal data for monitoring changes in sleep patterns and circadian rhythms over time. The potential of this smartphone app extends beyond insomnia research and could be applied to broader public health initiatives focused on understanding sleep health and mental well-being in the general population.

### Limitations

Several methodological limitations should be noted when interpreting this study’s findings. First, it is important to note that the “Rhythm” app was developed based on the Android operating system, and future efforts should be made to create versions compatible with other operating systems, such as iOS and Windows. Second, due to the small sample size used in the clinical correlations, caution is warranted when generalizing our algorithm’s performance, and further studies with larger and more diverse samples are necessary for validation. Third, there were certain challenges in capturing smartphone behaviors during physically inactive states (eg, WASO_app_ during actigraphy-defined sleep) and mental activities during physically active states (eg, sleep inertia in actigraphy-defined awakening but app-defined sleep). Future research could focus on addressing these aspects to enhance the accuracy and comprehensiveness of our data. Fourth, incorporating a weekly phone interview for actigraphy validation may be prone to recall bias, unlike the daily completion intended for diaries. Last, we acknowledge a potential limitation related to the precise definition of moments when users temporarily leave their smartphones, such as during work, charging, or other activities. The smartphone’s inactivity during these intervals presented difficulties in accurately capturing app use patterns. To overcome this limitation, improved data collection methods or additional sensors could be explored to provide more detailed insights into user interactions with their smartphones during such periods.

### Conclusion

In conclusion, this study addressed a significant gap in the literature by investigating circadian rhythm and sleep indicators in individuals with insomnia, an understudied population in smartphone-based sleep-wake cycle estimations. We introduced a novel approach of capturing circadian rhythms through human-smartphone interactions, complementing the traditional physical activity-based methods of actigraphy. The findings highlighted significant correlations between IV_app_ and depressive symptom scores, as well as RA_act_ and depressive symptom scores, aligning with patterns observed in individuals with depression. This study’s unique focus on patients with insomnia and innovative methodological approach offer valuable insights into the clinical relevance of smartphone-derived circadian rhythm indicators in mental health research. Additionally, the study demonstrated the potential of smartphone apps for digital health research and internet medical research, paving the way for broader applications in understanding sleep health and mental well-being in diverse populations. These strengths collectively contribute to advancing digital health research and offer opportunities for future investigations into the impact of sleep disruptions on mental health outcomes.

## Abbreviations

DSM-5: Diagnostic and Statistical Manual of Mental Disorders, Fifth Edition
IS: interdaily stability
IV: intradaily variability
IV_act_: actigraphy-based intradaily variability
IV_app_: app-defined intradaily variability
L5: least active 5 continuous hours
L5_app_: app-defined least active period
M10: most active 10 continuous hours
NAWK: number of awakenings
NAWK_app_: actigraphy-based number of awakenings
NAWK_app_: app-defined number of awakenings
PHQ-9: Patient Health Questionnaire
PSQI: Pittsburgh Sleep Quality Index
RA: relative amplitude
RA_act_: actigraphy-based relative amplitude
RA_app_: app-defined relative amplitude
SO: sleep onset
SO_act_: actigraphy-based sleep onset
SO_app_: app-defined sleep onset
TST: total sleep time
TST_act_: actigraphy-based total sleep time
TST_app_: app-defined total sleep time
WASO: wake after sleep onset
WASO_act_: actigraphy-based wake after sleep onset
WASO_app_: app-defined wake after sleep onset
WT: wake time
WT_act_: actigraphy-based wake time
WT_app_: app-defined wake time

## Data Availability

The data sets generated and analyzed during this study are not publicly available due to restrictions imposed by informed consent from participants in this study. This consent has been approved by the National Taiwan University Hospital and Chang-Gung Memorial Hospital Institutional Review Board. However, data can be made available to researchers who meet the criteria for access to confidential data through the Institutional Review Board of the NHRI. For those who wish to request access, the data can also be obtained from the corresponding author upon reasonable request.
